# Deregulated Levels of the NF-κB1, NF-κB2, and Rel Genes in Ukrainian Patients with Leukemia and Lymphoma in the Post-Chernobyl Period

**DOI:** 10.4274/tjh.2014.0190

**Published:** 2016-02-17

**Authors:** Hakan Savlı, Ramis Ufuk Akkoyunlu, Naci Çine, Daniil F. Gluzman, Michael P. Zavelevich, Lilia M. Sklyarenko, Stella V. Koval, Deniz Sünnetçi

**Affiliations:** 1 Kocaeli University Faculty of Medicine, Department of Medical Genetics, Kocaeli, Turkey; 2 National Academy of Sciences of Ukraine, R.E. Kavetsky Institute of Experimental Pathology, Oncology, and Radiobiology, Kyiv, Ukraine

**Keywords:** Chronic lymphocytic leukemia, non-Hodgkin’s lymphoma, B-Cell neoplasms, Cancer, Thrombosis, T-cell neoplasms, B-Cell neoplasms, Acute leukemia, Myelodysplastic syndromes, Chronic leukemia

## Abstract

**Objective:**

Nuclear factor kappa B (NF-κB) is an important transcription factor in cancer and NF-κB activation has been seen in angiogenesis, tumor progression, and metastasis. Relationships between specific NF-κB gene networks, leukemogenesis, and radiation exposure are still unknown. Our aim was to study the expression levels of the NF-κB1, NF-κB2, and Rel genes in hematological malignancies in the post-Chernobyl period.

**Materials and Methods:**

We analyzed gene expression levels of NF-κB1, NF-κB2, and Rel in 49 B-cell chronic lymphocytic leukemia, 8 B-cell non-Hodgkin’s lymphoma, 3 acute myeloid leukemia, 3 chronic myeloid leukemia, 2 hairy cell leukemia, 2 myelodysplastic syndrome, and 2 T-cell large granular lymphocytic leukemia patients using real-time polymerase chain reaction.

**Results:**

Expression levels of NF-κB1, NF-κB2, and Rel genes were found to be deregulated.

**Conclusion:**

These results could be accepted as specific gene traces to radiation-induced leukemia or as potential candidates for new diagnostic biomarker studies. Larger experiments and non-exposed control malignant cell populations are needed to clarify these suggestions.

## Abstract (tr)

**Amaç:**

Nükleer faktör kappa B (NF-κB), kanserde önemli bir transkripsiyon faktörü olup aktivasyonu anjiyogenez, tümör gelişimi ve metastazın birçok basamağında görülmektedir. Spesifik NF-κB gen ağları, lökomogenez ve radyasyon maruziyeti arasındaki ilişki halen belirsizdir. Çalışmamızda Çernobil sonrası hematolojik kanserlerde NF-κB1, NF-κB2 ve Rel genlerinin ekspresyon düzeylerini incelemeyi amaçladık.

**Gereç ve Yöntemler:**

Gerçek zamanlı polimeraz zincir reaksiyonu ile 49 B-hücreli kronik lenfositik lösemi, 8 B-hücreli non-Hodgkin lenfoma, 3 akut myeloid lösemi, 3 kronik myeloid lösemi, 2 tüylü hücre lösemi, 2 miyelodisplastik sendrom ve 2 T-hücreli büyük granüler lenfositik lösemi hastasında NF-κB1, NF-κB2 ve Rel gen ekspresyon düzeylerini analiz ettik.

**Bulgular:**

NF-κB1, NF-κB2 ve Rel genlerine ait ekspresyon düzeyleri değişmiş olarak saptandı.

**Sonuç:**

Bu sonuçlar, radyasyonla indüklenmiş lösemilerdeki spesifik gen izleri veya yeni tanısal biyobelirteç çalışmalarına muhtemel aday önerileri olarak da kabul edilebilir. Bu düşünceleri açıklığa kavuşturmak için daha geniş deneyler ve radyasyon maruziyeti olmayan kontrol malign hücre popülasyonlarına ihtiyaç vardır.

## INTRODUCTION

The derivations of signatures using proteomics and genomics are increasingly integrated in the design of prognostic and predictive markers in oncology. Some of these markers are also well-known targets for therapeutic approaches, such as bortezomib, a nuclear factor kappa B (NF-κB) inhibitor possessing clinical activity in mantle cell lymphoma patients [1]. NF-κB is an important transcription factor in immunity, cell proliferation, cell survival, and cancer [2,3,4,5]. NF-κB activation has been demonstrated in angiogenesis, tumor progression, and metastasis [6,7].

Relationships between gene networks, leukemogenesis, and radiation exposure are still unknown. Our aim was to study expression levels of the NF-κB1 gene family in Ukrainian B-cell chronic lymphocytic leukemia (B-CLL), B-cell non-Hodgkin’s lymphoma (NHL), acute myeloid leukemia (AML), myelodysplastic syndrome (MDS), chronic myeloid leukemia (CML), hairy cell leukemia (HCL), and T-cell large granular lymphocytic leukemia (T-cell LGLL) patients in the post-Chernobyl period.

## MATERIALS AND METHODS

Samplesof theperipheral bloodand bonemarrow of49 B-CLL,8 B-cell NHL,3 AML,3 CML,2 HCL,2 MDS,and 2T-cell LGLL patients were obtainedfrom the R.E. Kavetsky Institute of Experimental Pathology, Oncology, and Radiobiology of the National Academy of Sciences of Ukraine in 2008 and 2009. The mean age ofthe B-CLL groupwas 58.7 years and the median was 60 years (minimum 36 yrs, maximum 87 yrs). In theB-cell NHL group, the mean age was 57.3 years and the median was 60 years (minimum 43 yrs, maximum 69 yrs). Patients were analyzed morphologically and immunocytochemically according to the new World Health Organization classification, asshown in Table 1(Continuation-1-2-3) along with demographical data. Thecontrol group comprised the peripheral blood samples of 8 healthy donors from Ukraine. Themean age of the secontrol subjects was 45.9 years and the median was 42.5 years (minimum 27 yrs, maximum 78 yrs). All B-CLL cases under study were of the typical B-CLL immunophenotype without adverse prognostic markers such as CD38+. Total ribonucleic acid (RNA) was isolated from leukocytes using the QIAamp RNA Blood Mini Kit (QIAGEN, Valencia, CA, USA) and treated with DNase I according to the manufacturer’s instructions. Quantity and purity were checked using a NanoDrop 2000 UV-Vis Spectrophotometer (Thermo Scientific, Wilmington, DE, USA). Complementary deoxyribonucleic acid (cDNA) was synthesized using a RevertAid First Strand cDNA Synthesis Kit (Fermentas Inc., Hanover, MD, USA) from 100 ng/µL total RNA as starting material. Gene expression levels were determined by quantitative reverse transcription-polymerase chain reaction as described previously [8,9]. Standard curves were obtained using serial dilutions of the beta-globulin gene (DNA Control Kit, Roche, Penzberg, Germany). Gene-specific primers (Table 2) were obtained from Integrated DNA Technologies (Coralville, IA, USA). Obtained gene expression values were normalized using a housekeeping gene of beta-2 microglobulin. Gene expression ratios were compared in patient and control groups using the Relative Expression Software Tool (REST).

### Statistical Analysis

Statistical analysis was performed using independent sample t-tests to analyze the statistical significance of our results by comparing controls with B-CLL, B-cell NHL, AML, MDS, CML, HCL, and T-cell LGLL patients. The p-values are shown in Table 3.

## RESULTS

The NF-κB1, NF-κB2, and Rel genes were found to be upregulated in 49 B-CLL, 8 B-cell NHL, 3 AML, and 2 HCL patients in the post-Chernobyl period (Table 3). NF-κB1 was decreased 1.301-fold in B-CLL, 1.473-fold in B-cell NHL, 1.534-fold in AML, and 1.862-fold in HCL cases. NF-κB2 was upregulated 1.720-fold in B-CLL, 8.545-fold in B-cell NHL, 16.257-fold in AML, and 1.676-fold in HCL cases. We found Rel expression upregulated 2.736-fold in B-CLL, 4.039-fold in B-cell NHL, 65.526-fold in AML, and 6.912-fold in HCL cases.

In the MDS group, NF-κB2 was found to be significantly upregulated (50.563-fold). Rel was 2.272-fold upregulated whereas NF-κB1 was 1.100-fold downregulated in the same group.

In the CML group, NF-κB2 was 2.110-fold upregulated while NF-κB1 and Rel were downregulated 1.056-fold and 1.239-fold, respectively.

We found downregulation of the NF-κB1, NF-κB2, and Rel genes in T-cell LGLL cases at 4.557-fold, 3.771-fold, and 2.632-fold, respectively.

## DISCUSSION

We had already found deregulated levels of NF-κB in our genomic experiments on prostate cancer [10], papillary thyroid cancer [11], and leukemia [12,13] in our previous studies. Recently, our proteomic results confirmed the upregulation of NF-κB in microarray screening in a breast cancer population [14]. This is our first observation of NF-κB deregulations in hematopoietic malignancies.

Transcription of proteins that promote cell survival, stimulate growth, induce angiogenesis, and reduce susceptibility to apoptosis are upregulated by NF-κB. The NF-κB signaling pathway was found activated in MDS, AML, acute lymphoblastic leukemia (ALL), CML, CLL, multiple myeloma, and lymphoma cases before. These 3 genes were defined as deregulated before in hematological malignancies [15]. Here we have observed deregulated levels in radiation-induced leukemia populations. Results supported that radiation-exposed and non-exposed hematological malignancies use the same gene pathways and are shaped around the NF-κB gene network.

It was shown that losses in the 13q chromosomal region are also associated with B-CLL and these losses deregulate the NF-κB pathway [16]. Deregulation of the NF-κB pathway by gains at chromosomal loci including the NF-κB1, NF-κB2, and Rel genes was reported previously. A gain at the (2)(p16.1p14) region including the Rel gene, an oncogene, was reported in 17p-deleted CLL with poor prognosis [17]. Rearrangements such as translocations and deletions occurred in 10q24 affecting the NF-κB2 gene, a protooncogene. These rearrangements are known to lead to deletion of 3’ sequences of the NF-κB2 gene and cause production of carboxy-truncated constitutively nuclear proteins that may have a role in the tumorigenesis of B-CLL and B-cell NHL at high levels [18]. Unlike its relative NF-κB2, NF-κB1 has few rearrangements reported in leukemias and lymphomas. There is evidence in the literature that NF-κB2 is involved in oncogenesis in T-cell ALL as a result of LYL1 translocation [19]. Further studies are needed to assess the NF-κB1 rearrangements leading to B-CLL and B-cell NHLs. These observations give us new clues about relationships between NF-κB deregulation in leukemias and chromosomal regions. We are planning to continue our further studies by array comparative genomic hybridization analysis to focus on fine mapping of 13q and 2p in particular.

Over the last decade, the problem of association between B-CLL and ionizing radiation has become a matter of considerable scientific interest [20]. Nevertheless, the experimental studies on the relationship between ionizing radiation and CLL are limited. Lyng et al. indicated that activation of the NF-κB pathway may suppress the apoptotic response in U698 cells, a malignant B-lymphocyte cell line, to ionizing radiation [21]. Activation of the NF-κB pathway by ionizing radiation induces antiapoptotic genes and inhibits apoptosis by upregulation of NF-κB genes. This was linked to proliferation and increased survival of B-CLL [22]. B-CLL and HCL cells are known to be refractory to signals activating normal B cells. B-CLL and HCL cells are stimulated by tumor necrosis factor (TNF-α) [23]. TNF-α is involved in many human tumors and associated with poor prognosis. TNF-α is produced by B-CLL and HCL cells [24] and contributes to the escape of HCL cells from apoptosis through NF-κB activation [25]. Radiation exposure results in high levels of NF-κB gene expression. We found upregulated levels of NF-κB1, NF-κB2, and Rel genes in our patients. Our results were in concordance with the previous findings above.

NF-κB expressions were found significantly higher than in the controls in both AML and ALL by Kapelko-Słowik et al. before [26]. They also found lower expression levels of NF-κB in AML patients who reached complete remission compared with patients with primary resistance to chemotherapy who did not reach complete remission. These data indicated that high expression levels of NF-κB might be involved in the pathogenesis of AML and ALL [26]. There are few studies on radiation-induced leukemia populations, such as in the Chernobyl area. However, we cannot assess all etiological sources for our subjects. Levels of exposure among subjects during the Chernobyl accident remain unclear. Thus, we cannot conclude that the NF-κB pathway is the main cause of AML and ALL pathogenesis in radiation-induced forms of the disease.

Rel has the potential to transform cells in culture and is expressed in high levels in both B-cell NHL [27] and large granular lymphocytic leukemia [28]. Interestingly, we found decreased levels of Rel, NF-κB1, and NF-κB2 in our T-cell LGLL group.

In our study we found NF-κB2 significantly higher in MDS cases. There is evidence in the literature that the degree of NF-κB activity is correlated with the risk of progression to AML. NF-κB activation is known to be a hallmark of high-risk MDS [27].

We obtained increased levels of NF-κB2 in CML cases. Exposure to ionizing radiation causes CML [29]. CML is characterized by t(9;22), which leads to Bcr/Abl fusion oncoprotein expression. This protein activates the NF-κB pathway. The NF-κB pathway, in turn, leads to expression of antiapoptotic proteins such as Bcl-XL and lets Bcr/Abl+ cells grow [27].

Here we have defined a positive correlation between upregulated levels of NF-κB genes in hematological malignancies related to radiation exposure. However, the limited number of patients and controls was an obstacle. Therefore, these experiments are presented here as results of a preliminary study. Similar studies should be extended to experiments in time- and dose-dependent manners in cell lines or primary cultures. We think that our results are a good starting point for drawing a network around the NF-κB genes to investigate the life cycles of hematological malignancies.

## CONCLUSION

Moreover, it would be tempting to suggest that this gene region may be used as a trace of early radiation exposure leading to leukemia. Either way, the NF-κB pathway certainly deserves more attention since its overexpression is almost a rule in many solid tumors and hematopoietic malignancies.

### Ethics

Ethics Committee Approval: Bioethics Committee of the R.E. Kavetsky Institute of Experimental Pathology, Oncology and Radiobiology of the National Academy of Sciences of Ukraine (Approval number: 5/2008), Informed Consent: It was taken.

## Figures and Tables

**Table 1A:**
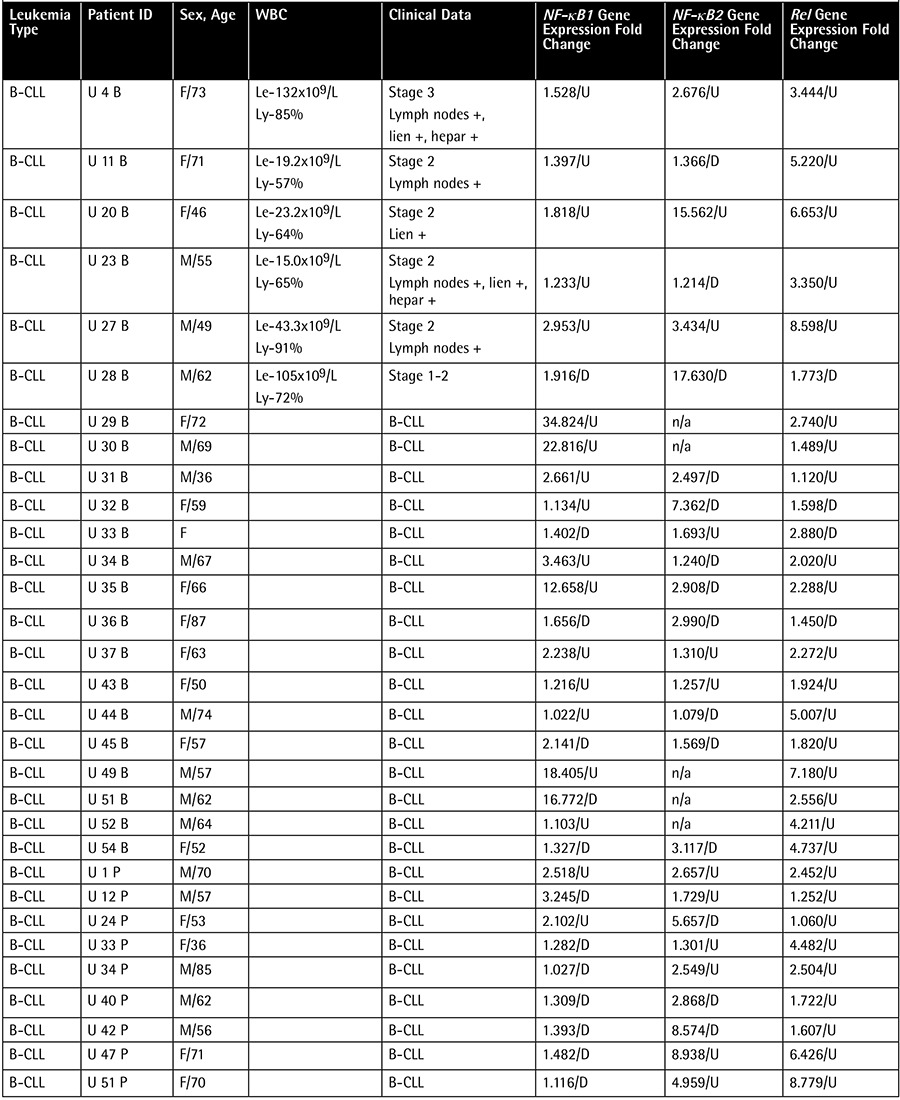
Patient data, clinical features, and individual gene expression ratios.

**Table 1B:**
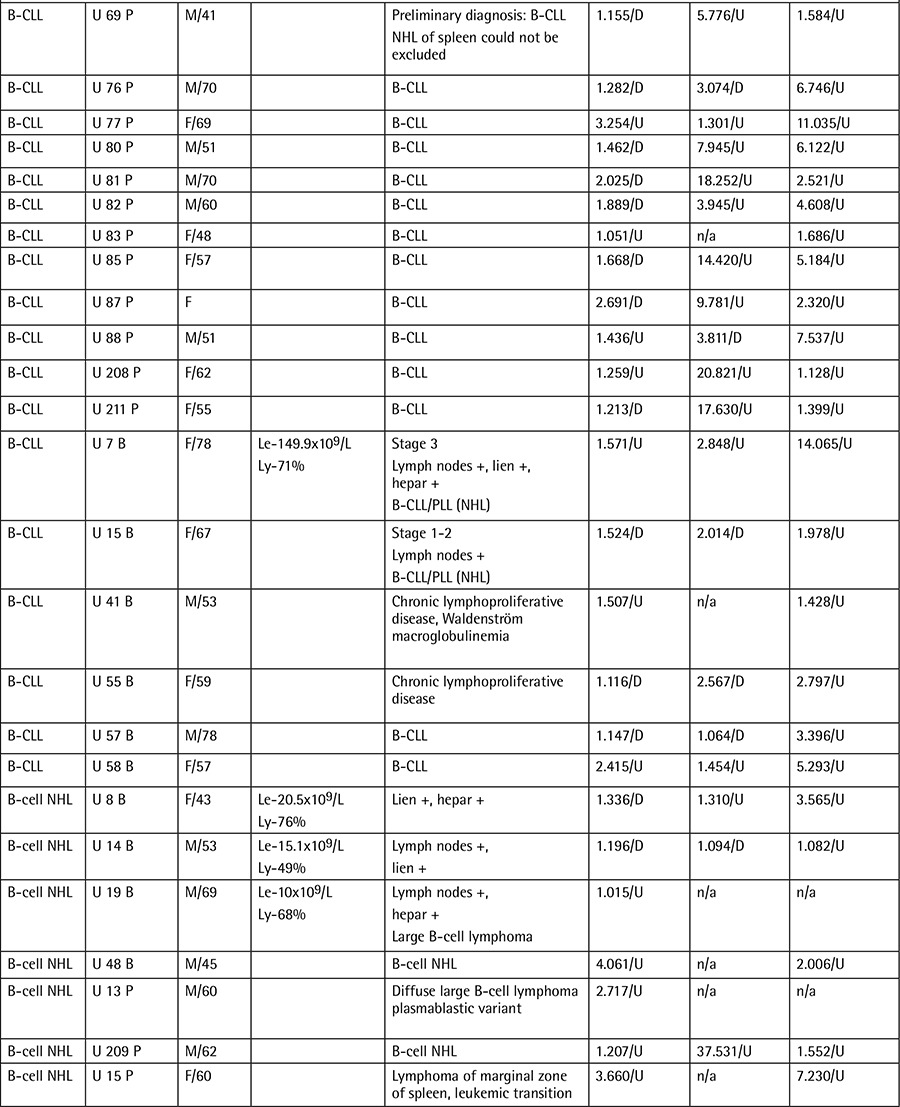
Patient data, clinical features, and individual gene expression ratios.

**Table 1C:**
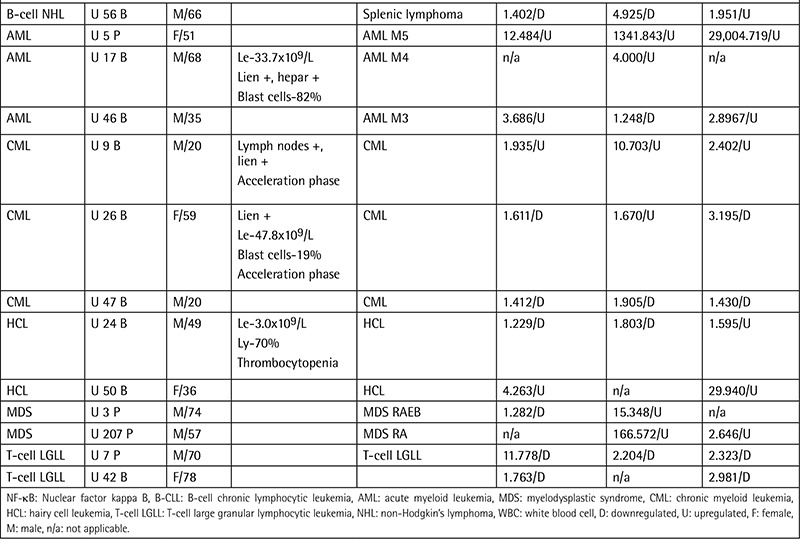
Patient data, clinical features, and individual gene expression ratios.

**Table 2:**
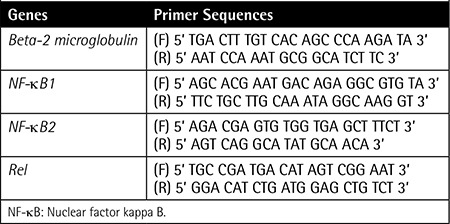
Primer sequences of the studied genes.

**Table 3:**
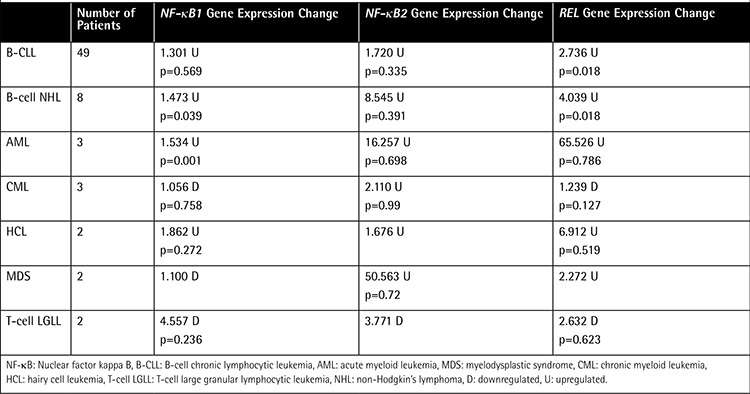
Gene expression levels in groups of studied patients.
